# Prevalence, Causes, and Risk Factors of Presenting Visual Impairment and Presenting Blindness in Adults Presenting to an Examination Center in Suzhou, China

**DOI:** 10.1155/2022/2885738

**Published:** 2022-12-20

**Authors:** Ruizhu Sun, Dan Huang, Zhenxing Liu, Tingting Zhu, Zheyao Gu, Ge Ma, Yun Wang, Chunyuan Zhang, Xiangying Luo, Zhigang Tang, Ting Xi, Fangfei Xie

**Affiliations:** ^1^Department of Ophthalmology, The Affiliated Suzhou Hospital of Nanjing Medical University, Suzhou Municipal Hospital, Gusu School, Nanjing Medical University, 242 Guangji Road, Suzhou, Jiangsu 215008, China; ^2^Physical Examination Center, The Affiliated Suzhou Hospital of Nanjing Medical University, Suzhou Municipal Hospital, Gusu School, Nanjing Medical University, 242 Guangji Road, Suzhou, Jiangsu 215008, China

## Abstract

**Purpose:**

To evaluate the prevalence, causes, and risk factors of presenting visual impairment (PVI) and presenting blindness among adults in Suzhou, China.

**Methods:**

A total of 43927 subjects were included in this cross-sectional study. Each subject underwent ophthalmic examinations, including presenting visual acuity (PVA), intraocular pressure (IOP), slit-lamp examination, and fundus examination under the small pupils of each eye.

**Results:**

Using the World Health Organization (WHO) definition, the prevalence of bilateral PVI, bilateral presenting blindness, monocular PVI, and monocular presenting blindness was 1.59% (95% CI, 1.51–1.67), 0.002% (95% CI, 0.0019–0.0021), 3.87% (95% CI, 3.68–4.06), and 0.19% (95% CI, 0.18–0.20), respectively. Using the United States (US) definition, the prevalence of bilateral PVI, bilateral presenting blindness, monocular PVI, and monocular presenting blindness was 5.83% (95% CI, 5.54–6.12), 0.04% (95% CI, 0.038–0.042), 7.43% (95% CI, 7.06–7.80), and 0.45% (95% CI, 0.43–0.47), respectively. The prevalence of PVI was higher in females (WHO criteria, 2.06%, 95% CI, 1.96–2.16; US criteria, 7.27%, 95% CI, 6.91–7.63) than in males (WHO criteria, 1.2%, 95 CI%, 1.14–1.26; US criteria, 4.65%, 95% CI, 4.42–4.89). The leading cause of PVI is an uncorrected refractive error, followed by cataracts and age-related macular degeneration (AMD). Multivariate analysis proved that the prevalence of visual impairment (PVA, better eye, WHO criteria) increased significantly with older age, higher mean arterial pressure (MAP), higher globulin level, and higher fasting blood glucose (FBG). In addition, it also increased significantly with lower hemoglobin, a lower body mass index (BMI), and a lower arterial stiffness index. In this study, serum creatinine, blood urea nitrogen, uric acid, triglycerides, and the systemic immune-inflammation index (SII) showed no association with visual impairment.

**Conclusion:**

The leading causes of PVI in Suzhou were uncorrected refractive error and cataracts. The prevalence of PVI increased with females, older age, higher MAP, higher FBG, higher globulin, lower hemoglobin, lower BMI, and lower arterial stiffness index.

## 1. Introduction

The WTO launched the World Report on Vision in New York in November 2019. According to the report, about 2.2 billion people worldwide were plagued by VI, or blindness [1]. These figures must be substantially increased due to population growth, aging [2], and lifestyle changes. By 2050, about 610 million people will suffer from blindness, 474 million will suffer from moderate or severe vision impairment, and 360 million will suffer from mild vision impairment [3]. Vision impairment and blindness have lasting effects on all aspects of life and affect people of all ages [4], including economic and educational opportunities [5], experience problems in mobility and usual activities [6], negative emotions like anxiety or depression [7], increased likelihood of falls and fractures [8], and a high risk of cognitive decline in older people [8]. The risk for all-cause death was higher in those with vision impairment, and the importance of this effect increased with more severe vision impairment [9].

According to the WTO report, at least 1 billion people with a visual impairment could have the opportunity to recover or improve their visual acuity [1]. First of all, the prevalence and causes of visual impairment should be clarified so that a more efficient and economic strategy can be developed to improve the visual acuity of the population. Numerous epidemiological studies have reported the prevalence and causes of VI in the Chinese population [10–13], and several systematic reviews and meta-analyses have assessed the VI prevalence in the Chinese population [14, 15]. However, China is the most populous country in the world and the third largest in terms of land area. The prevalence of vision impairment and blindness varied widely from study to study and across regions.

Suzhou is an economically developed city in Jiangsu Province. It borders Shanghai to the east, Zhejiang Province to the south, Taihu Lake to the west, and the Yangtze River to the north. Located in East China, southeast of Jiangsu Province, it is one of the important central cities in the Yangtze River Delta. There was a lack of data on visual impairment in all age groups in Suzhou. Our study managed to report the current prevalence, causes, and possible risk factors of PVI and presenting blindness in the adult population of Suzhou.

## 2. Methods

The methods used in this study is explained in the following sections.

### 2.1. Ethics Approval and Consent to Participate

The study complied with the Declaration of Helsinki. The Ethics Committee of The Affiliated Suzhou Hospital of Nanjing Medical University approved this study's protocol and informed consent forms (Ethical Approval Number: KL901223). Before coming to the physical examination center, all subjects received written notice and informed consent. Verbal consent was obtained from all subjects before the eye examination.

### 2.2. Study Population and Data Collection

This physical examination center-based, cross-sectional study was carried out from January 2021 to December 2021. This study used convenience sampling. All residents aged 18 years and above who visited the physical examination center during the study period were eligible to participate in the study. We excluded the following residents: (1) residents who refused to participate in this study; (2) nonlocal permanent residents; (3) those incapable of autonomous behavior due to cognitive impairment and other reasons; (4) those who refuse to accept an ophthalmological examination; (5) due to the participants' personal reasons or poor cooperation, they could not complete all the eye examinations; (6) stabilized vision cannot be obtained from diseases such as acute inflammation of the eye; and (7) those who have not completed any of the other examinations in this study, such as blood drawing, measuring height and weight, and so on. The Physical Examination Center of the Suzhou Hospital of Nanjing Medical University is the only statutory medical examination center established by the Suzhou Municipal Health Bureau. More than 100,000 people come for medical examinations every year. The physical examination population covers the whole city. The physical examination objects are mainly residents, including the annual physical examination of employees, the recruitment examination of enterprises and institutions, and the physical examination of people from all walks of life. In addition, it also includes government-funded physical examinations for elderly residents, students' physical examinations for high school and college entrance examinations, medical examinations for recruited national civil servants, and military enlistment medical examinations. After each patient receives the ophthalmic examination, we directly enter the data into the electronic system of the medical examination center. The data used in this study were retrieved from the electronic medical records of the Physical Examination Center of the Suzhou Hospital of Nanjing Medical University.

After registering the participants, we recorded their ages, genders, heights, and weights. We also collected the medical history of eye disease, presenting visual acuity (PVA) in both eyes, intraocular pressure (IOP), cause of visual impairment, blood routine, and blood biochemistry. Blood pressure was measured with an automated sphygmomanometer (HBP-9021, Omron Corp., Kyoto, Japan). Fasting blood samples were obtained via venipuncture and tested within half an hour. The computational formula of the mean arterial pressure (MAP) is

(1) $$\begin{array}{c} diastolic\;blood\;pressure+\frac{1}{3}pulse\;pressure\text{.} \end{array}$$

The computational formula of the body mass index (BMI) is

(2) $$\begin{array}{c} \frac{weight}{{height}^{2}}\text{.} \end{array}$$

The computational formula of the systemic immune-inflammation index (SII) is

(3) $$\begin{array}{c} platelet\;count\times \left(\frac{neutrophil}{lymphocyte}\right)\text{.} \end{array}$$

The computational formula of the arterial stiffness index is

(4) $$\begin{array}{c} \frac{cholesterol-high\;density\;lipoprotein}{high\;density\;lipoprotein}\text{.} \end{array}$$

We categorized subjects into six groups according to age: 18 to 29 years old, 30 to 39 years old, 40 to 49 years old, 50 to 59 years old, 60 to 69 years old, and 70 and older than 70 years. We defined age over 60 years as older adults.

### 2.3. Eye Examinations

All subjects were examined and diagnosed by the ophthalmologist. They came from the department of ophthalmology at the Affiliated Suzhou Hospital of Nanjing Medical University. They have more than three years of clinical experience and received standardized training before they worked for the Physical Examination Center. After entering the ophthalmic examination room, the subjects underwent an ocular screening examination, including bilateral assessment of PVA, IOP, slit-lamp examination, and a fundus examination with small pupils. The anterior segment of the eye was evaluated using a slit-lamp (YZ5G, 66 Vision Tech Co., Ltd., Suzhou, China). The posterior segment was examined using a fundoscopy lens (90D, Volk Optical Inc., Ohio, USA) at the slit lamp. We also measured the IOP with a noncontact tonometer (FT-1000, Topcon, Inc., Tokyo, Japan) and recorded the average of the three readings.

PVA was measured and recorded separately in each eye with the tumbling E standard logarithmic visual chart light box (Product Standard GB1533-2011, Hualong Medical, China) at a 3 m distance. Subjects wearing habitual spectacles were recorded as having corrected visual acuity, and those who did not wear spectacles were recorded as having uncorrected visual acuity. Examinations were conducted in a room with standardized illumination and no direct sunlight or shadows. If the subject fails to recognize the top line at 3 m, move to 2 m and 1 m. If no letter could be read, VA was recorded as counting fingers, hand movements, light perception, or no perception of light. If PVA was worse than 20/40, we measured the best corrected visual acuity with a spherical lens without cycloplegia. First, we put the −1.00 DS spherical lens in front of the subject to check whether the corrected visual acuity is improved. If the visual acuity improves, put −1.00 DS, −1.50 DS, −2.00 DS, −2.50 DS, −3.00 DS, and other spherical lenses in front of the patient's eyes, respectively, until the best corrected visual acuity of the patient is achieved. The ophthalmologist recorded the corrected visual acuity and spherical powers of the patients. If −1.00 DS cannot improve corrected visual acuity, we put a +1.00 DS spherical lens in front of the patient to check whether the correct visual acuity improves. If there is no improvement in corrected visual acuity, record the patient's visual acuity and note that the corrected visual acuity does not improve. If the +1.00 DS spherical lens improves the corrected visual acuity of the participant, put the +1.00 DS, +1.50 DS, +2.00 DS, +2.50 DS, +3.00 DS, and other spherical lenses in front of the patient in sequence until the patient achieves the best corrected visual acuity. Then the ophthalmologist recorded the patients' best corrected visual acuity and spherical power.

### 2.4. Definition of PVI and Presenting Blindness

We used two criteria to define PVI and presenting blindness in our study. According to the WHO criterion, bilateral PVI was defined as PVA < 20/63 to 20/400 in the better eye, and bilateral presenting blindness was defined as PVA < 20/400 in the better eye [16]. According to the US criterion, bilateral PVI was defined as PVA < 20/40 to 20/200 in the better eye, and bilateral presenting blindness was defined as PVA < 20/200 in the better eye [14]. We also presented data on monocular PVI and monocular presenting blindness.

### 2.5. Causes of PVI and Presenting Blindness

The principal causes of PVI and presenting blindness were determined by examining ophthalmologists. If the subject has multiple ophthalmic problems, the untreatable disease causing VI is considered the primary cause. We recorded the leading cause of visual impairment when PVA < 20/40. We regarded cataracts as the cause of VI if there was no evidence of corneal opacity, vitreous hemorrhage, or retinopathy in an eye with significant lens opacity. The lens status was recorded as intraocular lens or aphakia if the subject had undergone cataract surgery. We diagnosed uncorrected refractive error if the eyes improved to ≥20/32 with refractive correction. The diagnostic criteria for myopic maculopathy were spherical refraction greater than −6.00D in either eye with typical degenerative myopic fundus changes, such as patchy chorioretinal atrophy, macular atrophy, or choroidal neovascularization. Age-related macular degeneration (AMD) was diagnosed based on characteristic fundus manifestations, including macular area drusen and atrophy, subfoveal disciform scarring, and exudative or neovascular macular degeneration. Since this study did not include a visual field examination and gonioscopy, glaucoma was suspected if the subject had the following symptoms: a shallow anterior chamber, a cup-to-disc ratio(C/D) ≥0.6 in either eye, or a C/D asymmetry ≥0.2, and IOP >21 mmHg. Elevated intraocular pressure alone was not a diagnostic criterion for glaucoma. We defined diabetic retinopathy (DR) in diabetic subjects with hard exudates, cotton wool spots, intraretinal hemorrhage, and microaneurysms. We defined corneal opacity by the presence of nebula, corneal macula, leukoma, or corneal pannus. We defined other causes of VI according to routine clinical diagnosis and the medical history of eye disease. Subjects whose eye examination showed no organic disease were recorded as having nothing abnormal detected. All subjects with suspected eye diseases were referred to the ophthalmology clinic for further investigation.

### 2.6. Statistical Analysis

Statistical analysis was conducted using SPSS Statistics (version 17.0, IBM/SPSS, Inc., USA). The chi-square test was used to analyze the prevalence of PVI and presenting blindness among different gender groups. Univariate and multivariate logistic regression analyses were used to analyze the possible risk factors of PVI. *P* values less than 0.05 were considered statistically significant.

## 3. Results

The results obtained are described in the following sections.

### 3.1. Prevalence of Presenting Visual Impairment and Presenting Blindness

A total of 43927 subjects (24139, 54.95% male) from 1 July 2021 to 30 December 2021 were included in the study. Their mean age was 52 ± 16 years (range 18–97 years). In this study, we used crude prevalence rates and did not adjust for age, but we stratified the subjects by age and counted the prevalence of blindness and visual impairment at different ages. Using the WHO standard, the prevalence of bilateral PVI and bilateral presenting blindness was 1.59% and 0.002%, respectively. The prevalence of monocular PVI and monocular presenting blindness was 3.87% and 0.19%, respectively (Table 1). Using the US standard, the prevalence of bilateral PVI and bilateral presenting blindness was 5.83% and 0.04%, respectively. The prevalence of monocular PVI and bilateral presenting blindness was 7.43% and 0.45%, respectively (Table 2). The prevalence of bilateral and monocular PVI was higher in the 70+ age group when compared with the younger and middle age groups (Tables 1 and 2). The rate of PVI among women was higher than that among men, both according to WHO (*P* < 0.01) and US criteria (*P* < 0.01). However, the prevalence of presenting blindness showed no association with gender (*P* > 0.05).

### 3.2. Causes of Presenting Visual Impairment and Presenting Blindness

We have summarized the causes of bilateral PVI according to WTO and US definitions in Table 3. The leading causes of bilateral PVI were uncorrected refractive error, cataracts, and AMD, whether by WTO or US definition. Besides, we divided subjects into 18–39 years old, 40–60 years old, and 60+ years old. The causes of bilateral PVI were analyzed separately in each group. We found that uncorrected refractive error was the leading cause in young and middle-aged groups (18–60 years old), while cataract was the leading cause in the elder age group (60+ years old), both with WHO and US standards (Table 3).

**Figure 1:**
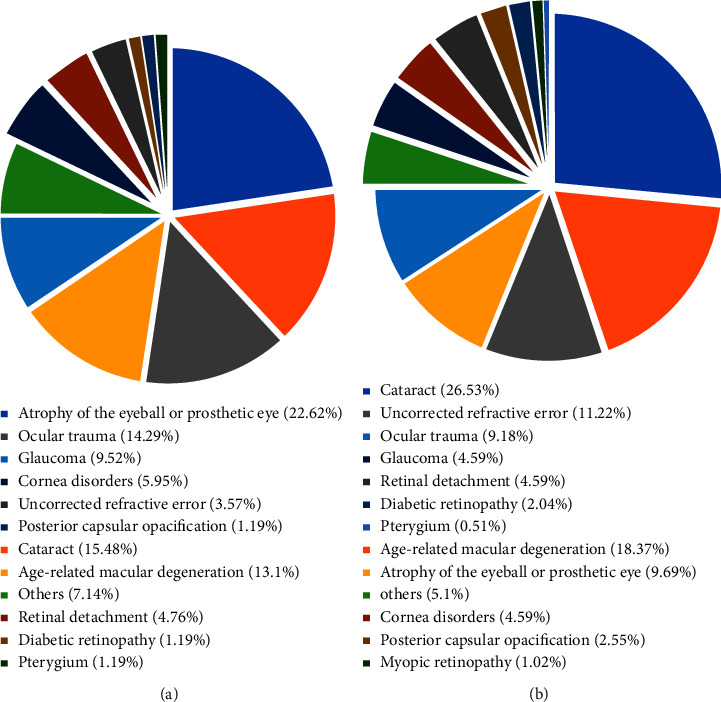
The distribution of monocular presenting blindness causes using the WTO and US standards. (a) Monocular presenting blindness causes using the WTO standard; (b) monocular presenting blindness causes using the US standard.

There were 18 subjects detected with bilateral presenting blindness using US criteria. The causes were cataracts (9 subjects, 50%), uncorrected refractive error (4 subjects, 22.22%), DR (3 subjects, 16.67%), AMD (1 subject, 5.56%), and cornea disorders (1 subject, 5.56%). According to WTO standards, one subject was detected with bilateral presenting blindness. The main cause was bilateral cataracts. Using WTO criteria, the primary causes of monocular presenting blindness were atrophy of the eyeball or prosthetic eye, cataract, ocular trauma, and AMD in our study (Figure 1(a)). According to US criteria, the principal causes of monocular presenting blindness were cataract, AMD, and uncorrected refractive error (Figure 1(b)).

### 3.3. Associated Factors for Presenting Visual Impairment and Presenting Blindness

This study analyzed some possible risk factors of visual impairment using WHO criteria, including age, BMI, MAP, arterial stiffness index, triglycerides, hemoglobin, FBG, uric acid, blood urea nitrogen, serum creatinine, SII, and globulin (Table 4). We also showed in Table 4 the number of subjects in different ranges for the variables we analyzed. Univariate analysis showed that the prevalence of bilateral PVI using WHO criteria was significantly associated with age, MAP, hemoglobin, arterial stiffness index, triglycerides, BMI, FBG, blood urea nitrogen, uric acid, SII, and globulin (all *P* < 0.05). On multivariable analysis, the prevalence of bilateral PVI was significantly associated with older age, higher MAP, higher FBG, higher globulin level, lower hemoglobin level, lower BMI, and lower arterial stiffness index.

## 4. Discussion

Our study assessed the prevalence, causes, and possible risk factors of visual impairment and blindness in adults with a mean age of 52 years (range 18–97) in Suzhou City of Jiangsu Province, a district in eastern China. PVA was used to assess visual impairment in our study, which better reflected the real-life situation and more accurately assessed the burden of visual impairment than best-corrected visual acuity. Differences in study populations, designs, and definitions should be considered compared with previous studies.

In this study, we observed that the occurrence of PVI and presenting blindness increased significantly with older age, especially in adults aged 70 years and older. This result may be due to the higher incidence of age-related eye disease in the elderly, leading to limited improvement in corrected visual acuity in older adults. According to WTO criteria, bilateral and monocular vision impairment prevalence was 1.59% and 3.87%, respectively. Moreover, bilateral and monocular blindness were 0.002% and 0.19%, respectively. Using US criteria, the prevalence of bilateral and monocular vision impairment was 5.83% and 7.43%, respectively. Furthermore, the prevalence of bilateral and monocular blindness was 0.04% and 0.45%, respectively. Compared with other studies, the prevalence of PVI and presenting blindness in this study was lower than in China and other countries [11, 13, 14, 17–19]. The potential reasons may be the discrepancy in investigation samples, survey methods, age composition, and socioeconomic situation among different studies. This study obtained research samples from the Suzhou physical examination center. Suzhou is a relatively developed city, and the subjects of this study lived in the urban district of Suzhou. They voluntarily come to the physical examination at their own expense, or their employer pays for the health check-up. They may have a more stable income and be more health-conscious. When they have a vision impairment, they may be more aggressive in seeking treatment options. On the plus side, better economic status and health awareness may reduce the occurrence of visual impairment to a certain extent. Additionally, we found higher visual impairment feasibility in women than in men. This finding was in accordance with previous research [3, 20].

We found that uncorrected refractive error was the leading cause of PVI, followed by cataracts, AMD, myopic retinopathy, DR, and other eye diseases. These findings were similar to those of previous studies [17, 19]. Indeed, on a global scale, uncorrected refractive error is the leading cause of PVI. A systematic review identified 288 studies that showed that the leading causes of visual impairment were uncorrected refractive error, cataract, AMD, glaucoma, and DR [2]. Another meta-analysis of East Asia showed that uncorrected refractive error was the leading cause of vision impairment and the second cause of blindness [21]. We have calculated the proportion of oculopathy in different age groups. The results showed that the leading cause of PVI in the young and middle-aged (18–60 years) was uncorrected refractive error. For adults aged 60 years and older, cataract was the leading cause of PVI. Since refractive error and cataracts cause most of the visual impairment and blindness and can be reversed with spectacle correction (refractive errors) and surgery (cataracts), governments and medical organizations can develop programs to increase the rate of eyeglass wear and cataract surgery. In a global context, the prevalence of myopia and high myopia has grown remarkably in the past few years [22], especially in East Asia [23, 24]. The occurrence and development of high myopia could cause a series of complications, such as myopic retinopathy and retinal detachment, which can lead to blindness. In the present survey, myopic retinopathy was the fourth leading cause of PVI. Myopia develops in childhood and aggravates with age. The incidence of high myopia increased with the incidence of myopia. Protecting the visual health of adolescents is a top priority.

This study analyzed some systemic risk factors contributing to visual impairment and blindness. We found that the risk of visual impairment increased significantly with increased MAP, FBG, and decreased hemoglobin. In this study, serum creatinine, blood urea nitrogen, and triglyceride levels were not associated with PVI. Clinically, arteriosclerosis, hyperglycemia, hypertension, and hyperlipidemia are the causes of various retinopathy and optic nerve diseases, such as retinal vein occlusion, diabetic retinopathy, retinal artery occlusion, arteriosclerotic retinopathy, hypertensive retinopathy, and ischemic optic neuropathy. Anemia caused by reduced hemoglobin may lead to ischemia and hypoxia in ocular tissues, which may cause optic nerve and retina-related diseases. All of these eye diseases might cause irreversible visual impairment. The Hong Kong Eye Study has studied some systemic risk factors associated with vision impairment, such as BMI, cholesterol, triglycerides, HDL, LDL, and creatinine, but the results showed that they were not significantly associated with vision impairment [19]. A previous study found that Hba1c, triglycerides, total cholesterol, LDL/HDL ratio, and blood pressures were significantly higher in DR with DME but no significant changes in blood urea and serum creatinine [25]. A study on AMD found that lipid status disorders and inflammation may play an essential role in the development of AMD in older adults [26]. Another study showed that the risk of AMD increased significantly with a higher body mass index, whereas blood pressure, HbA1c, and blood lipid-associated factors were not associated with AMD [27]. Aortic stiffness has been a risk factor for DR and peripheral neuropathy in diabetic patients [28]. However, our study found that a lower body mass index and a lower arterial stiffness index were the risk factors for visual impairment, which was difficult to interpret. These different conclusions require further research to verify them. Serum globulins are a mixture of various proteins, including immunoglobulins, complements, and glycoproteins. Elevated serum globulins are commonly associated with chronic infectious diseases (like chronic liver disease), autoimmune diseases (like rheumatoid diseases), malignant diseases (like cancer), the nephrotic syndrome, and diabetes mellitus [29]. The present study found serum globulin to be a risk factor for PVI and blindness, which was not mentioned in previous studies. The specific pathogenesis is unclear, but it may be related to immune-related eye diseases (like uveitis) or ocular complications of systemic diseases (like diabetic retinopathy). The specific mechanism needs further study. Recently, several studies have shown that an elevated serum uric acid (SUA) level is a risk factor for diabetic retinopathy [30, 31]. In addition, inflammation plays a vital role in diabetic retinopathy [32], and a recent study found that the systemic immune-inflammation index may be a diagnostic biomarker for diabetic macular edema [33]. In this study, we also analyzed serum uric acid and the systemic immune-inflammation index, but they were not significantly associated with vision impairment and blindness. The causes and risk factors for visual impairment are diverse and complex. These results contradict previous studies, and more research may be needed to investigate. The strengths of the present study include a wide age range of participants, a large sample size, and an analysis of risk factors associated with vision impairment.

Limitations of this cross-sectional study include the following points: first, the subjects of the current research underwent physical examinations voluntarily. They have to pay for their expenses for the medical examination. Therefore, to some extent, they may have higher incomes or pay more attention to their physical health than other residents. That probably led to a selection bias. Second, 87.99% of the subjects were younger than 60, 46.44% were younger than 40, and only 4.72% were 70 or older. Therefore, the overall prevalence of the subjects may be underestimated. However, we stratified subjects by age and calculated the prevalence of visual impairment by age. Thirdly, this study did not use some ophthalmic equipment, such as optical coherence tomography and perimeter, which may lead to misdiagnosis or missed diagnosis of eye diseases like DR, glaucoma, and AMD. Fourth, this study was a cross-sectional study. We assessed the influence of various risk factors at only one point in time.

## 5. Conclusions

Our study confirmed that the leading causes of PVI in Suzhou were uncorrected refractive error and cataracts, both of which were curable. That reminded us that the prevalence of presenting visual impairment might decrease if we increase the rate of spectacle wearing and cataract surgery. The prevalence of PVI increased with age and was higher in women than men. In addition, the prevalence of PVI might be related to blood glucose, blood pressure, serum globulin, and hemoglobin. Further studies are needed to carefully evaluate the role of biologically relevant risk factors contributing to visual impairment and blindness in adults.

## Figures and Tables

**Table 1:** Prevalence of presenting visual impairment and presenting blindness (World Health Organization definition) stratified by age and gender.

Group (y)	No. of subjects	Bilateral				Monocular			
		Visual impairment		Blindness		Visual impairment		Blindness	
		No.	% (95% CI)	No.	%	No.	% (95% CI)	No.	% (95% CI)
*Male*									
18–29	3250	27	0.83 (0.79–0.87)	0	0	73	2.25 (2.14–2.36)	2	0.06 (0.06–0.06)
30–39	6924	36	0.52 (0.49–0.55)	0	0	139	2.01 (1.91–2.11)	2	0.03 (0.03–0.03)
40–49	5535	46	0.83 (0.79–0.87)	0	0	133	2.40 (2.28–2.52)	5	0.09 (0.09–0.09)
50–59	5365	56	1.04 (0.99–1.09)	0	0	194	3.62 (3.44–3.80)	11	0.21 (0.20–0.22)
60–69	1730	23	1.33 (1.26–1.40)	0	0	105	6.07 (5.77–6.37)	10	0.58 (0.55–0.61)
70+	1335	102	7.64 (7.26–8.02)	0	0	126	9.44 (8.97–9.91)	28	2.1 (2.0–2.21)
Total	24139	290	1.20 (1.14–1.26)	0	0	770	3.19 (3.03–3.35)	58	0.24 (0.23–0.25)

*Female*									
18–29	3604	62	1.72 (1.63–1.81)	0	0	150	4.16 (3.95–4.37)	0	0
30–39	6623	89	1.34 (1.27–1.41)	0	0	211	3.19 (3.03–3.35)	2	0.03 (0.03–0.03)
40–49	4347	73	1.68 (1.60–1.76)	0	0	150	3.45 (3.28–3.62)	3	0.07 (0.07–0.07)
50–59	3004	64	2.13 (2.02–2.24)	0	0	119	3.96 (3.76–4.16)	9	0.3 (0.29–0.32)
60–69	1385	41	2.96 (2.81–3.11)	0	0	113	8.16 (7.75–8.57)	3	0.22 (0.21–0.23)
70+	825	78	9.45 (8.98–9.92)	1	0.12	189	22.91 (21.76–24.06)	9	1.09 (1.04–1.14)
Total	19788	407	2.06 (1.96–2.16)	1	0.005	932	4.71 (4.47–4.95)	26	0.13 (0.12–0.14)

*Combined*									
18–29	6854	89	1.30 (1.24–1.37)	0	0	223	3.25 (3.09–3.41)	2	0.03 (0.03–0.03)
30–39	13547	125	0.92 (0.87–0.97)	0	0	350	2.58 (2.45–2.71)	4	0.03 (0.03–0.03)
40–49	9882	119	1.20 (1.14–1.26)	0	0	283	2.86 (2.72–3.00)	8	0.08 (0.08–0.08)
50–59	8369	120	1.43 (1.36–1.50)	0	0	313	3.74 (3.55–3.93)	20	0.24 (0.23–0.25)
60–69	3115	64	2.05 (1.95–2.15)	0	0	218	7.00 (6.65–7.35)	13	0.42 (0.40–0.44)
70+	2160	180	8.33 (7.91–8.75)	1	0.05	315	14.58 (13.85–15.31)	37	1.71 (1.62–1.80)
Total	43927	697	1.59 (1.51–1.67)	1	0.002	1702	3.87 (3.68–4.06)	84	0.19 (0.18–0.20)

**Table 2:** Prevalence of presenting visual impairment and presenting blindness (United States definition) stratified by age and gender.

Group (y)	No. of subjects	Bilateral				Monocular			
		Visual impairment		Blindness		Visual impairment		Blindness	
		No.	% (95% CI)	No.	%	No.	% (95% CI)	No.	% (95% CI)
*Male*									
18–29	3250	106	3.26 (3.10–3.42)	0	0	129	3.97 (3.77–4.17)	5	0.15 (0.14–0.16)
30–39	6924	188	2.72 (2.58–2.86)	0	0	295	4.26 (4.05–4.47)	5	0.07 (0.07–0.07)
40–49	5535	196	3.54 (3.36–3.72)	1	0.02	297	5.37 (5.10–5.64)	11	0.2 (0.19–0.21)
50–59	5365	230	4.29 (4.08–4.50)	0	0	419	7.81 (7.42–8.20)	24	0.45 (0.43–0.47)
60–69	1730	110	6.36 (6.04–6.68)	2	0.12	219	12.66 (12.03–13.29)	23	1.33 (1.26–1.40)
70+	1335	293	21.95 (20.85–23.05)	7	0.52	285	21.35 (20.28–22.42)	48	3.6 (3.42–3.78)
Total	24139	1123	4.65 (4.42–4.89)	10	0.04	1644	6.81 (6.47–7.15)	116	0.48 (0.46–0.50)

*Female*									
18–29	3604	178	4.94 (4.69–5.19)	0	0	238	6.6 (6.27–6.93)	5	0.14 (0.13–0.15)
30–39	6623	356	5.38 (5.11–5.65)	0	0	378	5.71 (5.42–6.00)	8	0.12 (0.11–0.13)
40–49	4347	266	6.12 (5.81–6.43)	2	0.05	289	6.65 (6.32–6.98)	7	0.16 (0.15–0.17)
50–59	3004	224	7.46 (7.09–7.83)	1	0.03	300	9.99 (9.49–10.49)	17	0.57 (0.54–0.60)
60–69	1385	167	12.06 (11.46–12.66)	3	0.22	220	15.88 (15.09–16.67)	19	1.37 (1.30–1.44)
70+	825	247	29.94 (28.44–31.44)	2	0.24	195	23.64 (22.46–24.82)	24	2.91 (2.76–3.06)
Total	19788	1438	7.27 (6.91–7.63)	8	0.04	1620	8.19 (7.78–8.60)	80	0.4 (0.38–0.42)

*Combined*									
18–29	6854	284	4.14 (3.93–4.35)	0	0	367	5.35 (5.08–5.62)	10	0.15 (0.14–0.16)
30–39	13547	544	4.02 (3.82–4.22)	0	0	673	4.97 (4.72–5.22)	13	0.1 (0.10–0.11)
40–49	9882	462	4.68 (4.45–4.91)	3	0.03	586	5.93 (5.63–6.23)	18	0.18 (0.17–0.19)
50–59	8369	454	5.42 (5.15–5.69)	1	0.01	719	8.59 (8.16–9.02)	41	0.49 (0.47–0.51)
60–69	3115	277	8.89 (8.45–9.33)	5	0.16	439	14.09 (13.39–14.79)	42	1.35 (1.28–1.42)
70+	2160	540	25.00 (23.75–26.25)	9	0.42	480	22.22 (21.11–23.33)	72	3.33 (3.16–3.50)
Total	43927	2561	5.83 (5.54–6.12)	18	0.04	3264	7.43 (7.06–7.80)	196	0.45 (0.43–0.47)

**Table 3:** Causes for bilateral presenting visual impairment according to WTO and US definitions stratified by age.

Principal cause	WTO definition					US definition			
	18–39	40–59	60+	Total		18–39	40–59	60+	Total
	No. (%)	No. (%)	No. (%)	No. (%)		No. (%)	No. (%)	No. (%)	No. (%)
Uncorrected refractive error	212 (99.07)	147 (61.51)	2 (0.82)		361 (51.79)	822 (99.28)	594 (64.85)	2 (0.24)	1418 (55.73)
Cataract	0	8 (3.35)	198 (81.15)		206 (29.56)	0	10 (1.09)	666 (81.52)	676 (26.4)
Age-related macular degeneration	0	10 (4.18)	16 (6.56)		26 (3.73)	0	29 (3.17)	56 (6.85)	85 (3.32)
Myopic retinopathy	1 (0.47)	13 (5.44)	4 (1.64)		18 (2.58)	2 (0.24)	38 (4.15)	14 (1.71)	54 (2.11)
Diabetic retinopathy	0	7 (2.93)	5 (2.05)		12 (1.72)	0	24 (2.62)	10 (1.22)	34 (1.33)
Posterior capsular opacification	0	0	6 (2.46)		6 (0.86)	1 (0.12)	2 (0.22)	23 (2.82)	26 (1.02)
Glaucoma	0	2 (0.84)	4 (1.64)		6 (0.86)	0	11 (1.20)	9 (1.10)	20 (0.78)
Pterygium	0	3 (1.26)	1 (0.41)		4 (0.57)	1 (0.12)	13 (1.42)	5 (0.61)	19 (0.74)
Cornea disorders	0	1 (0.42)	4 (1.64)		5 (0.72)	0	4 (0.44)	7 (0.86)	11 (0.43)
Others	1 (0.47)	12 (5.02)	0		13 (1.87)	2 (0.24)	15 (1.64)	1 (0.12)	18 (0.70)
Undetermined	0	36 (15.06)	4 (1.64)		40 (5.74)	0	176 (19.21)	24 (2.94)	200 (7.81)
Total	214 (100)	239 (100)	244 (100)		697 (100)	828 (100)	916 (100)	817 (100)	2561 (100)

WTO: World Health Organization; US: United States.

**Table 4:** Logistic regression analysis of associations of bilateral vision Impairment (20/400 ≤ presenting visual acuity < 20/60, better eye).

Risk factors	No. of subjects			Multivariate analysis	Univariate analysis		
	Below normal	Normal range	Above normal	*P* value	OR	95% CI of OR	*P* value
Age	—	—	—	≤0.001	1.713	1.63–1.80	≤0.001
BMI	47	355	295	≤0.001	0.934	0.89–0.98	≤0.001
MAP	12	542	143	≤0.001	1.008	0.96–1.06	0.016
Arterial stiffness index	—	619	78	≤0.001	0.875	0.83–0.92	0.015
Triglycerides	1	559	137	≤0.001	1.045	0.99–1.10	0.915
Hemoglobin	195	491	11	≤0.001	0.987	0.94–1.04	≤0.001
FBG	0	562	135	≤0.001	1.058	1.01–1.11	0.042
Serum creatinine	128	555	14	0.644	—	—	—
Blood urea nitrogen	34	632	31	≤0.001	1.045	0.99–1.10	0.091
Uric acid	37	564	96	≤0.001	0.999	0.95–1.05	0.064
SII	—	278	419	0.037	1.000	0.95–1.05	0.006
Globulin	0	687	10	≤0.001	1.031	0.98–1.08	0.002

Below normal: the number of bilateral vision impairment subjects with low levels of the variable being analyzed. Normal range: the number of bilateral vision impairment subjects within the normal range for the variable being analyzed. Above normal: the number of bilateral vision impairment subjects with a high level of the analyzed variable. OR: odds ratio; 95% CI: 95% confidence interval; BMI: body mass index; MAP: mean arterial blood pressure; FBG: fasting blood glucose; SII: systemic immune-inflammation index.

## Data Availability

The datasets generated and analyzed during the current study are not publicly available due to subject privacy being involved but are available from the corresponding author upon reasonable request.
